# Focal HDR brachytherapy boost to stereotactic radiotherapy (fBTsRT) for prostate cancer: a phase II randomized controlled trial

**DOI:** 10.1186/s13014-022-02173-5

**Published:** 2022-12-09

**Authors:** C. Belliveau, M. Barkati, G. Delouya, D. Taussky, M. C. Beauchemin, C. Lambert, L. Beaulieu, D. Beliveau-Nadeau, B. Nicolas, J. F. Carrier, E. Vigneault, C. Ménard

**Affiliations:** 1 Radiation Oncology, CHUM - Centre Hospitalier de l’Université de Montréal, 900 Saint Denis St, Montreal, QC H2X 0A9 Canada; 2 Radiation Oncology, Centre universitaire de Québec, Université Laval, 2705 Laurier Boulevard, Quebec City, QC G1V 4G2 Canada

**Keywords:** Prostate cancer, Stereotactic radiotherapy, Focal brachytherapy, Quality of life, Magnetic resonance imaging

## Abstract

**Background:**

For patients with a higher burden of localized prostate cancer, radiation dose escalation with brachytherapy boosts have improved cancer control outcomes at the cost of urinary toxicity. We hypothesize that a focal approach to brachytherapy boosts targeting only grossly visualized tumor volumes (GTV) combined with stereotactic radiotherapy will improve quality of life (QoL) outcomes without compromising cancer control.

**Methods:**

150 patients with intermediate or high-risk prostate cancer will be enrolled and randomized 1:1 in a cohort multiple randomized clinical trial phase 2 design. Patients are eligible if planned for standard-of-care (SOC) high dose rate (HDR) brachytherapy boost to radiotherapy (RT) with GTVs encompassing < 50% of the prostate gland. Those randomly selected will be offered the experimental treatment, consisting of focal HDR brachytherapy boost (fBT) of 13–15 Gy in 1 fraction followed by stereotactic radiotherapy (sRT) 36.25-40 Gy in 5 fractions to the prostate (+/− 25 Gy to the elective pelvis) delivered every other day. The primary endpoint is to determine if fBTsRT is superior to SOC by having fewer patients experience a minimally important decline (MID) in urinary function as measured by EPIC-26 at 1 and 2 years. Secondary endpoints include rates of toxicity measured by Common Terminology Criteria for Adverse Events (CTCAE), and failure-free survival outcomes.

**Discussion:**

This study will determine whether a novel approach for the treatment of localized prostate cancer, fBTsRT, improves QoL and merits further evaluation.

*Trial registration* This trial was prospectively registered in ClinicalTrials.gov as NCT04100174 as a companion to registry NCT03378856 on September 24, 2019.

## Background

Prostate cancer is currently the most diagnosed cancer among men, accounting for approximately 20% of all malignancies found in this population [1]. Evolving techniques in radiotherapy (RT) for patients with intermediate or high-risk localized disease, including brachytherapy, image-guided radiotherapy (IGRT), intensity-modulated radiotherapy (IMRT), stereotactic radiotherapy (sRT), and most recently dose-painted radiotherapy, or a combination of these approaches with or without androgen deprivation therapy (ADT) have achieved ever-improving failure-free survival outcomes, especially in the context of dose escalation [2]. However, gains in cancer control achieved with the highest form of dose-escalation, brachytherapy boost, has come at a cost of added urinary toxicities as well as a decline in quality of life [3]. There is a need to explore strategies to reduce the rate of such toxicities, without compromising the gains achieved in local control outcomes.

Prostate cancer has been described as a multifocal disease [4], however visible tumors remain a strong predictor of biochemical failure free survival [5– 7]. Although recurrences almost always occur in the original site of dominancy, whole gland brachytherapy remains the standard of care. Modulating dose to grossly visualized tumor volumes (GTV) within the prostate gland has been shown to improve tumor control probability in modeling studies [8]. However, purported benefits are reduced when tumor targets are unnecessarily large, underscoring the importance of accuracy and precision for this approach [8]. More recently, the FLAME phase III trial provided the first clinical evidence of merit to a tumor-targeted approach, whereby a concomitant boost to GTVs up to 95 Gy (equivalent dose in 2 Gy/fraction [EQD2] 115 Gy, α/β ratio of 1.4) improved biochemical disease-free survival (bDFS) in comparison to standard of care (SOC) homogeneous RT 77 Gy (EQD2 82 Gy) in patients with intermediate to high-risk prostate cancer [2]. Moreover, extreme hypofractionation, such as sRT, has shown non-inferiority to standard fractionations in addition to benefiting from a radiobiological advantage for prostate cancer, all while having good tolerability and being cost-effective [9]. Strategies currently being investigated in dose escalating GTVs with integrated boost to sRT aim to deliver an EQD2 of 165 Gy (50 Gy/5 fractions) [10, 11].

Magnetic resonance imaging (MRI) can now be integrated in the brachytherapy workflow, either through direct online guidance [12] or computational image registration [13], supporting a focal tumor-targeted brachytherapy paradigm. In this trial we investigate the merits of this approach and hypothesize that focal brachytherapy combined with stereotactic radiotherapy will reduce urinary toxicity and improve quality of life in men with intermediate or high-risk prostate cancer when compared with SOC whole-gland high dose rate (HDR) brachytherapy boost to RT.

## Methods/design

### Objective

The objective of this trial is to determine if focal HDR brachytherapy boost to stereotactic radiotherapy (fBTsRT) is superior to SOC whole-gland HDR boost to RT in terms of urinary toxicity by having fewer patients experience a minimally important decline in urinary function.

### Study design

**Fig. 1:**
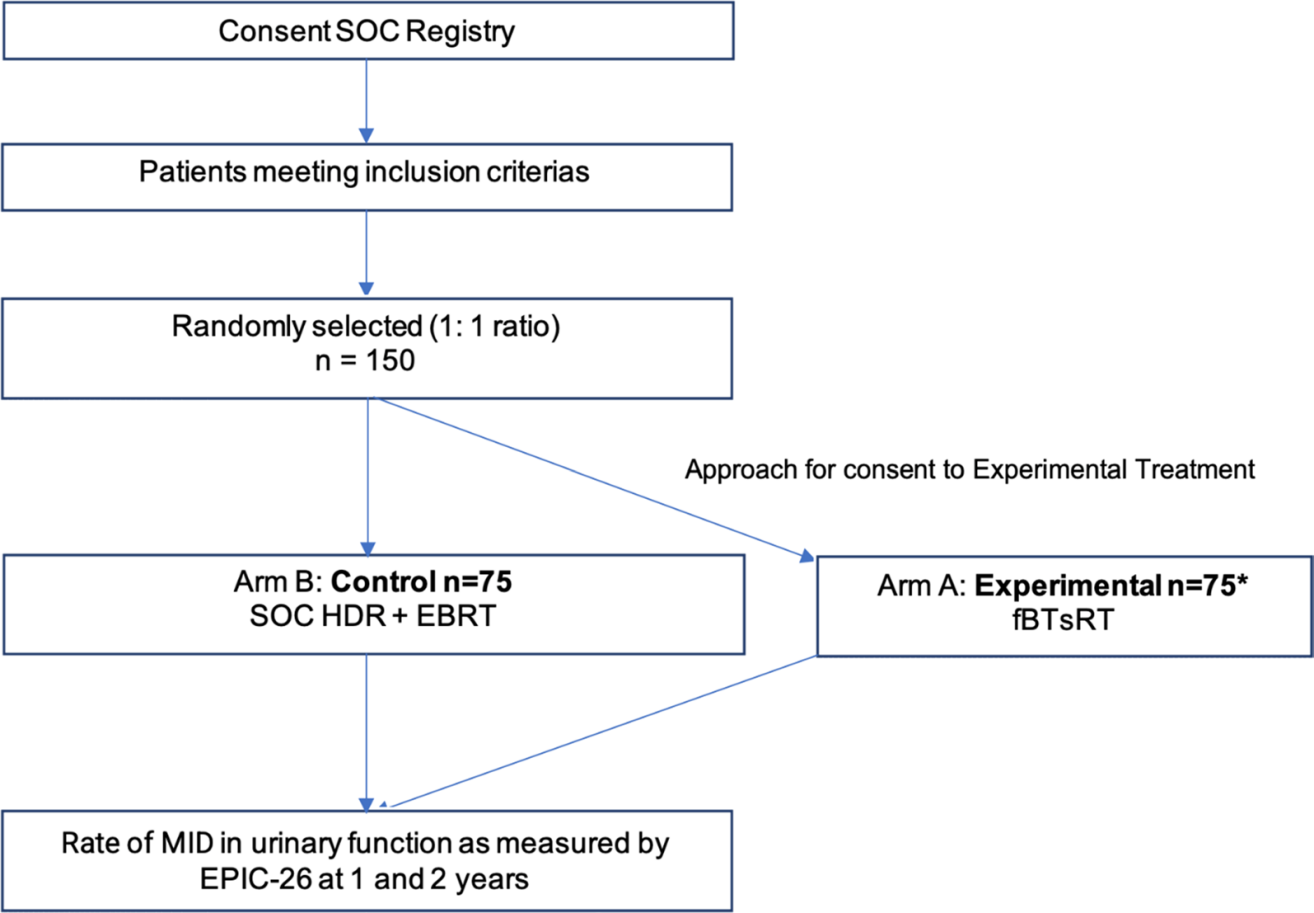
Study design

This is a pragmatic phase II cohort multiple randomized clinical trial (cmRCT) embedded into an existing prospective registry. Patients will be randomly selected (1:1) (Fig. 1), to be offered the experimental intervention, a focal HDR boost (fBT) of 13–15 Gy in 1 fraction followed by sRT 36.25-40 Gy in 5 fractions to the prostate clinical target volume (CTV) (± 25 Gy to the elective pelvis) delivered every other day in order to achieve a GTV EQD2 of 165 Gy. Patients assigned to the control cohort will receive SOC whole-gland HDR brachytherapy boost of 15 Gy in 1 fraction (± integrated GTV 18.75 Gy) followed by RT EQD2 43-47 Gy (5–22 fractions) to the prostate ± to the elective pelvis. Patients will be enrolled from two academic centers with an active prostate HDR brachytherapy program.

### Primary endpoint

The primary endpoint is the rate of minimally important decline (MID) in the urinary domain as measured Expanded Prostate Cancer Index Composite (EPIC)-26 at 12 and 24 months [14]. 

### Secondary endpoints

#### The secondary endpoints are as follow:

- Quality of life measures using EPIC-26. QLQC30, and EQ-5D-5L
- Rates of toxicity event measured by Common Terminology Criteria for Adverse Events CTCAEv5
- Rates of biochemical failure, defined as nadir + 2 ng/ml [15]. 
- Radiation dose metrics for targets and organs at risk (OARs)

### Eligibility criteria

#### The inclusion criteria are as follow:

- Patients with intermediate or high risk for prostate cancer according to National Comprehensive Cancer Network (NCCN) guidelines
- Histological diagnosis of prostate cancer planned for curative intent HDR brachytherapy boost to external beam radiotherapy.
- Eastern Cooperative Oncology Group (ECOG) 0–1
  - Charlson Comorbidity Index ≤ 5 [16]. 
  - MRI and/or Prostate-Specific Membrane Antigen-Positron Emission Tomography (PSMA-PET) visible disease encompassing < 50% of the prostate gland volume.
  - Enrolled in the PERa registry (Partnership for the Evaluation of Innovation in Radiotherapy -NCT03378856) and randomly selected to be offered experimental intervention.

### Exclusion criteria

#### The non-inclusion criteria are as follow:

- There are no exclusions

### Treatment planning and delivery

#### Experimental: fBTsRT

The experimental treatment consists of fBT boost of 13-15 Gy in 1 fraction prior to sRT.

**Fig. 2:**
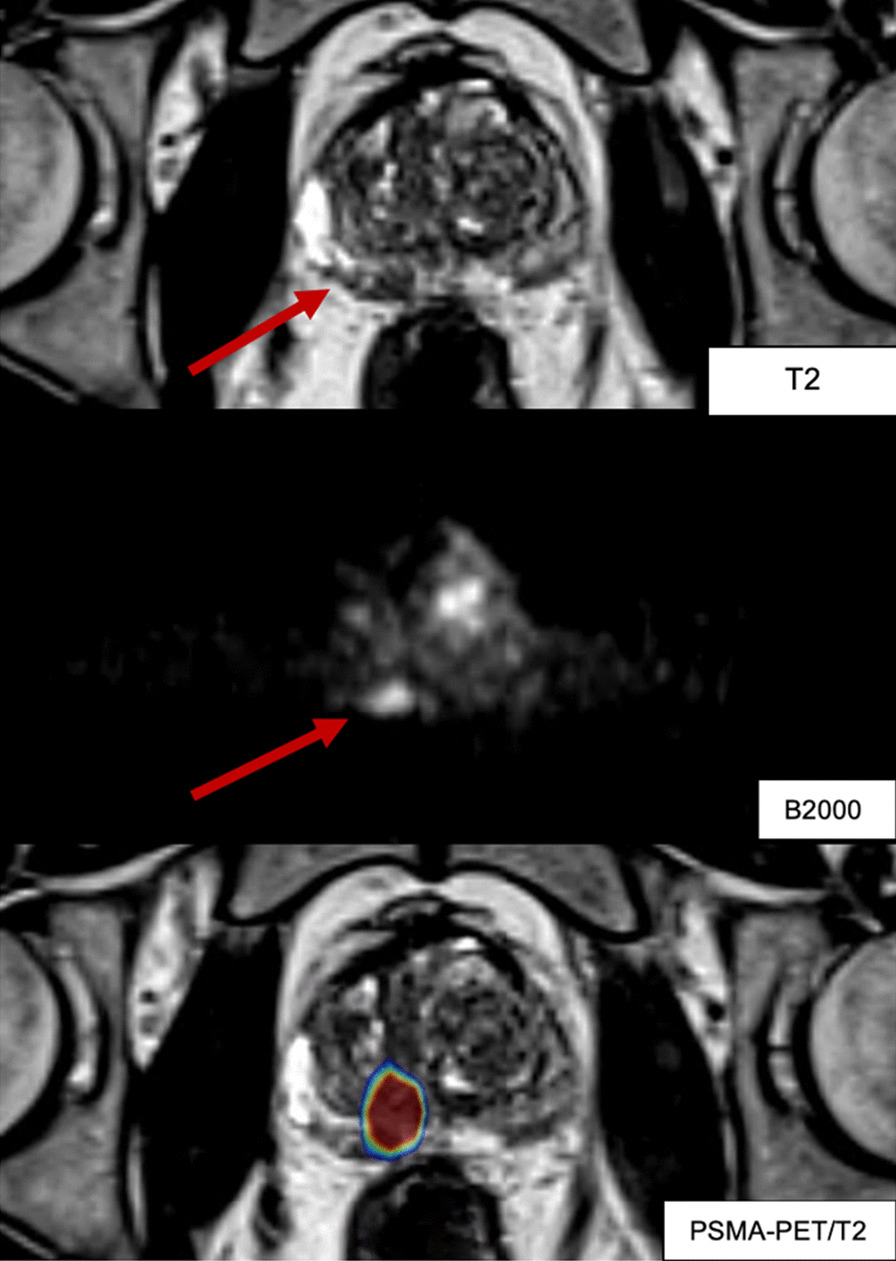
Images in T2, b2000 and PSMA-PET (PSMA-PET/CT registered to T2) images showing a GTV in the right peripheral zone of a patient planned for fBT

Patients will undergo MRI ((T2, b2000 DWI, ADC, ± DCE) and/or PSMA PET-CT (^18^F-DFCPyL), shown in Fig. 2, for treatment planning prior to the brachytherapy intervention for GTV segmentation. This data is then computationally integrated during the brachytherapy procedure [13]. HDR brachytherapy catheters are to be inserted within GTVs, and a 2 mm margin of uncertainty (3 mm SI) constrained to the prostate boundary will be applied to GTV targets for a final PTV. In cases with multiple tumors encompassing less than 50% of the prostate gland, catheters will be navigated within each GTV to achieve PTV coverage while respecting OAR constraints. Dose planning objectives are as follows: PTV V100% > 95%, rectal V75% < 0.5 cc, and urethral V125% < 0.1 cc.

sRT will follow fBT within 14 days. The dose prescribed in 36.25–40 Gy in 5 fractions to CTV with/without 25 Gy in 5 fractions to the elective pelvis, delivered every other day. The prostate gland plus 2 mm beyond GTV (restricted to the rectal boundary), and 1 cm beyond the GTV extending within the seminal vesicles will be delineated as the clinical target volume (CTV). Planning target volume (PTV) margins will be per SOC (3-5 mm). Dose planning objectives are as follows: PTV V36.25 Gy ≥ 95%, CTV V36.25-40 Gy ≥ 95%. See Table 1 for the risk-tolerance doses.

**Table 1:** sRT Organ at Risk Constraints

(Gy)	5 fractions		15 fractions		20 fractions		22 fractions	
	Major*	Minor**	Major*	Minor**	Major*	Minor**	Major*	Minor**
*Rectum*								
D50%	18	16	37	33	40	36	41	37
D25%	25	23	50	45	54	49	56	51
D15%	28	26	55	50	58	55	63	57
D0.5 cc	40	37	57	56	60	59	65	64
*Bladder*								
D2cc	39	38	39	35	42	38	43	39
D50%	18	16	50	46	55	50	57	52
D25%	24	21	56	52	60	57	64	59
D15%	28	25						
*Bowel Bag*								
D200cc	25	24	37	34	40	37	42	38
D3cc	29	26	43	38	47	42	48	43
D0.1 cc	38	34	49	44	54	48	56	50
*Femoral heads*								
D1%	28	22	42	32	46	34	47	35

^*^Major: Considered a planning priority (PTV compromised to respect coverage)

^**^Minor: Considered achieving without compromising PTV coverage

#### SOC whole-gland HDR brachytherapy boost RT

**Fig. 3:**
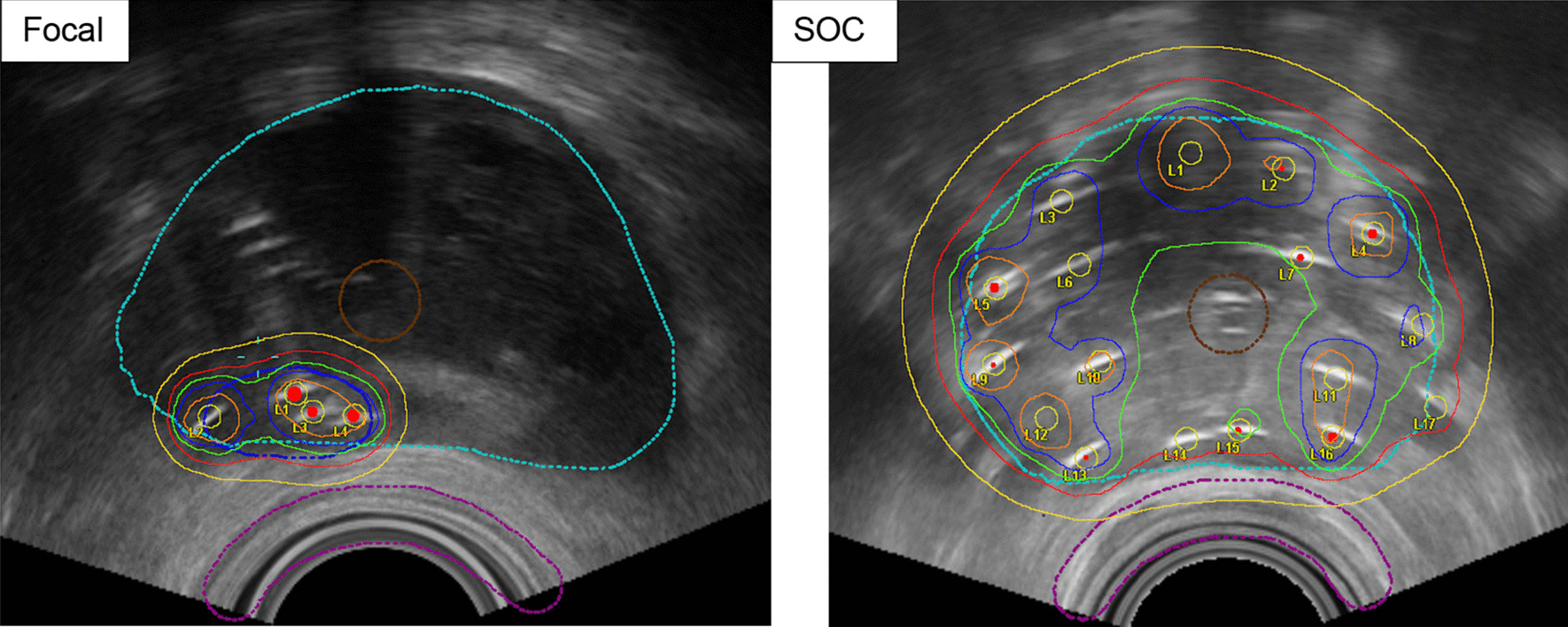
Comparison of fBT (4 catheters within the GTV-dark blue dotted line) and SOC HDR brachytherapy (17 catheters covering the prostate-light blue dotted line). Isodoses: Orange 200%; Dark Blue 150%; Green 125%; Red 100%; Yellow 75%. Organs: Prostate in light blue, Urethra in brown, Rectum in purple

The treatment in the control arm consists of standard of care offered to patients presenting with intermediate to high-risk prostate cancer. HDR brachytherapy will consist of 15 Gy to the prostate gland and visible extraprostatic disease (if present), with/without integrated GTV dose escalation to 18.75 Gy (125%), and delivered in one fraction. Dose planning objectives: V100% > 95% urethral Dmax < 118%, bladder and rectal Dmax < 80% of the prescription dose. Comparison of focal brachytherapy and SOC brachytherapy dose plan is shown in Fig. 3.

RT follows within 14 days with IMRT or Volumetric Modulated Arc Therapy (VMAT) techniques per SOC to an EQD2 43-47 Gy in 5–22 fractions to the prostate CTV ± elective pelvis.

**Fig. 4:**
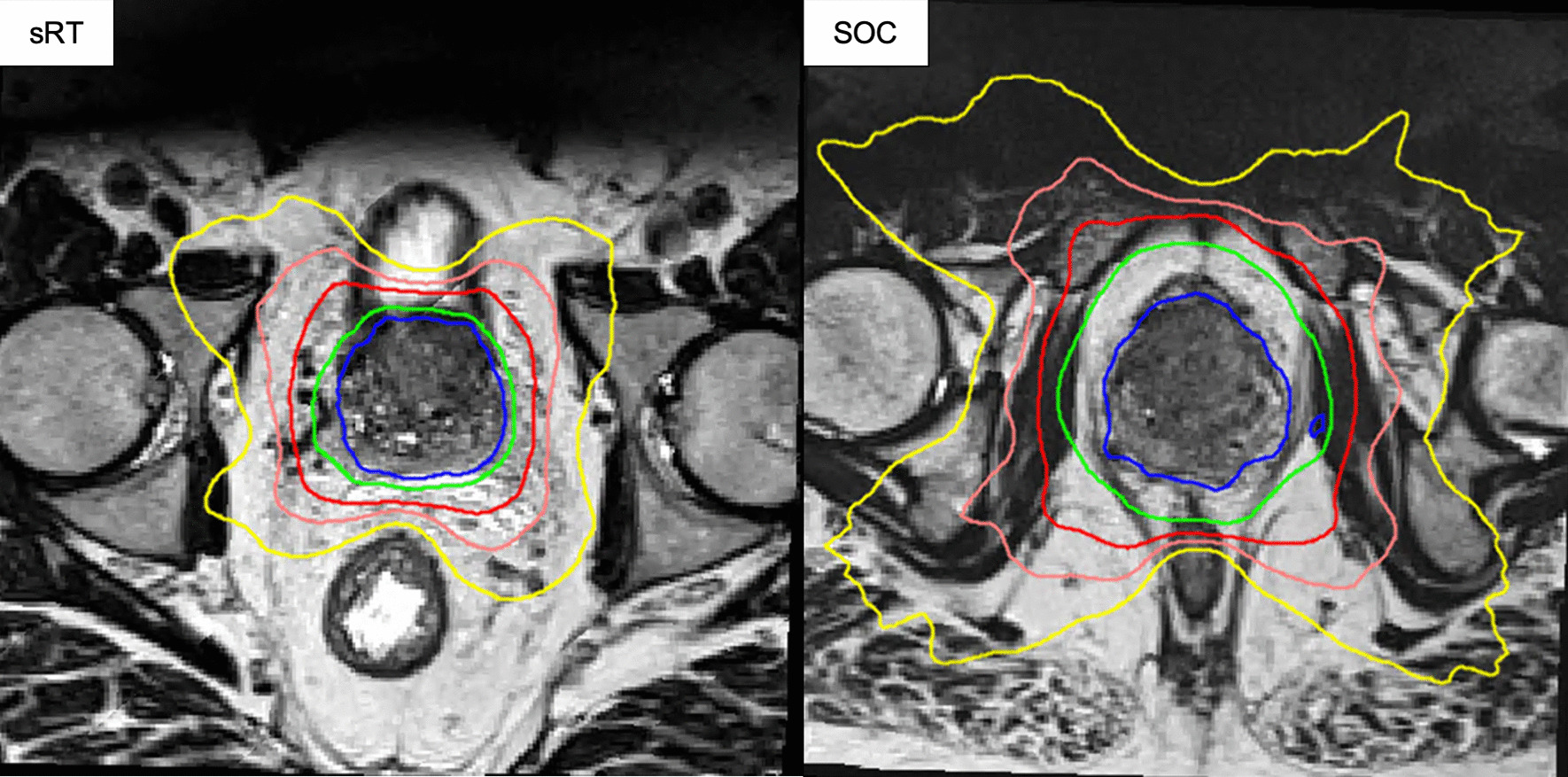
Isodose comparison of sRT and SOC RT on T2 image. Patient shown receiving sRT (Fig. 4) is the same as in Fig. 2 planned for fBT (Fig. 3). Isodoses: Blue 100%; Green 95%; Red 75%; Pink 60%; Yellow 45%

Case examples comparing sRT and SOC RT is shown in Fig. 4.

#### Androgen deprivation therapy (ADT)

ADT will be given per standard of care, and generally recommended. A change in use or duration of ADT from SOC is not permitted on this trial.

### Follow up evaluation

Patients will be followed per standard of care. At a minimum, baseline and annual evaluation with prostate-specific antigen (PSA), clinical evaluation for toxicity events, and quality of life (QoL) surveys will be completed. Subsequent staging investigations for suspected failure are to the discretion of the treating physician.

## Discussion

This phase II randomized trial seeks to investigate if fBTsRT is superior to standard care HDR brachytherapy boost to RT in terms of urinary toxicity by having fewer patients experience MID in urinary QoL.

With a novel approach to tumor dose escalation, we expect that patients will report fewer urinary symptoms for two main reasons. Focal targeting has the advantage of reducing the quantity of needle trauma to the prostate gland in comparison to whole gland HDR brachytherapy. This intervention is made possible by the work of our team and many others developing MRI-only or MRI-to-transrectal ultrasound (TRUS) registration for tumor-targeted prostate brachytherapy. Furthermore, by using focal brachytherapy techniques and sRT, dose escalation is maintained while limiting dose exposures to nearby OARs, therefore potentially limiting radiotherapy toxicities.

Oncological outcomes for prostate cancer patients are still dissatisfying in terms of unfavorable toxicities. While de-escalation has its uncertainties, individualized and risk-adapted treatments for patients with focal lesions are needed to achieve greater quality of life results all while preserving an excellent cancer control rate.

Prior studies have shown that patients treated with modern sRT have a significantly better quality of life when compared to patients treated with HDR brachytherapy boost to RT [17, 18] all while maintaining excellent long-term survival [9]. Recent FLAME study confirmed dose escalation to GTVs of up to EQD2 115 Gy improved bDFS in comparison to standard of care EBRT without any change in quality of life [2]. Even though concomitant boosts to GTVs has recently been showed superior, physicians are limited by the rectal dose constraints for posterior located tumors in RT. To our knowledge, no study has evaluated the efficacy of boosting GTVs with HDR. For this reason, focal brachytherapy as an alternative circumvents these rectal dose limits permitting dose escalation to GTVs in those regions.

As a logical evolution in the tumor-targeting paradigm in prostate cancer, this trial investigates a novel approach to better optimize the therapeutic ratio: achieving high cancer control while limiting toxicity and impact on quality of life.

## Data Availability

Data sharing is not applicable to this article as no datasets were yet generated or analyzed during this ongoing study.
